# Exploring the Role of the Behavioral Immune System in Acceptability of Entomophagy Using Semantic Associations and Food-Related Attitudes

**DOI:** 10.3389/fnut.2020.00066

**Published:** 2020-05-20

**Authors:** Kun Qian, Yuki Yamada

**Affiliations:** ^1^Institute of Decision Science for a Sustainable Society, Kyushu University, Fukuoka, Japan; ^2^Faculty of Arts and Science, Kyushu University, Fukuoka, Japan

**Keywords:** edible insect, insect food, disgust, eating behavior, food hygiene, eating insects, cognitive process, emotion

## Abstract

Entomophagy refers to eating insects. Insect food, including cooked insects and other processed food with insect-based ingredients, is consumed in many regions of the world as a part of traditional dietary behavior, or as innovative functional food. However, many people especially in western or industrialized societies have shown negative attitudes such as resistance or disgust to entomophagy. In this study, we examined the acceptability of eating insects from the context of the behavioral immune system (BIS), by employing a questionnaire survey and picture-based semantic association experiment. We collected data from 1,369 Japanese participants (581 females and 788 males, mean age = 43.41 years, *SD* = 10.44 years) by conducting an online survey. The results revealed the influence of the behavioral immune system on entomophagy: The semantic associations between insect food and non-insect food, and between insect food and pathogens were significantly predicted by multiple domains related to the attitudes, concerns, and experience of food and pathogens. The semantic associations between insect food and pathogens were significantly stronger than other associations. People who concentrate on food safety and hygiene revealed fewer associations between insect food and non-insect food. These results indicated that promoting the sanitary and hygienic image of insect food may reduce revulsion at entomophagy and promote uptake.

## Introduction

Entomophagy, which refers to the behavior of consuming insects as food and/or nutrients, is part of the dietary behavior of individuals in many countries and regions in the world especially in Africa, Asia, and Latin America (1, 2). More than 2,000 species of insects are consumed by more than 3,000 ethnic groups in 130 countries (3). However, insect food, referring to cooked insects or processed food with insect-based ingredients in this study, was found to be unacceptable by most individuals in so-called Western societies including Europe, North America, and Australia (2, 4). Cross-cultural comparative studies have revealed the different attitudes to eating insects between eastern and western cultures (5), and among 13 countries of six continents (6). However, in countries such as Cameroon (7), India (8, 9), and China (10, 11), where entomophagy has been a traditional dietary behavior, the consumption of insects has declined. In contrast, in western countries such as Australia (12), Hungary (13), Belgium (4), and the Netherlands (14), an increasing number of individuals have started to show interest or positive attitude to insect food. Exposure to entomophagy, such as providing information about or opportunity to try insect food, was found to contribute to positive attitudes toward insect food in some of these countries (15). In Japan, where we conducted the present study, the entomophagy has generally declined, but still exists as traditional food in some inland regions (16). Grasshoppers (*Oxya yezoensis* or *O. japonica*) and wasps (*Vespula* and *Dolichovespula* spp.) are the most popular insects consumed in Japan (17).

The Food and Agriculture Organization (FAO) has predicted that the global population will increase to nine billion in 2050. It is expected this will be accompanied by a significant increase in global food demand of up to 70% in comparison to current food requirements (18). Insect food is now considered and promoted as an ideal alternative source of protein because of its high environmental safety, and sustainable and efficient production (19). Besides the merits of food production, insect food constitutes a nutritionally balanced diet in comparison to traditional protein sources such as meat and eggs (20). Recent studies suggested that insect food is environmentally friendly because of less greenhouse gas emissions (21), and was nutritionally preferable to meat because of highly diverse nutritional composition (22). However, even though there are many advantages associated with and reasons to promote entomophagy, many individuals, especially westerners, have displayed doubt, resistance, and/or disgust to eating insects (23). Previous research has been conducted to explore the reasons thereof. La Barbera et al. (24), in their study on the role of food neophobia and implicit associations, found that changing implicit attitudes to edible insects could reduce reactions of disgust. Besides implicit associations, communication on the benefits of eating insects has affected attitude and eating behaviors (25). In most western countries, food neophobia has been viewed as the greatest deterrent associated with consuming insect food (13, 26). The roles of sensory-liking and food appropriateness were also examined in the context of eating insect food (27). Compared with processed insect foods, unprocessed insect foods were perceived as more unacceptable because of living food contaminant disgust (28). Experimental investigations demonstrated processing method and price-based quality inference as factors to manipulate the preference for insect food (29, 30). Familiarity and individual traits have also been found to influence the willingness to try insect food (31).

Research on aspects of human behavior has been conducted. The theory of planned behavior, which posits that individuals' behavioral intentions and behaviors are formed by their attitude to behavior, subjective norms, and perceived behavioral control, has been employed to explain the consumption of insect food (32). Factors of consumer behavior such as product attributes, official recommendations, and shopping locations were also revealed to be influential in consumer preferences for insect food (33). In the present study, the function of the behavioral immune system (BIS) in relation to the acceptability of eating insect food was examined. The BIS is a psychological mechanism that detects the potential existence of pathogenic parasites and accordingly, engages in behavior that prevents an individual from being exposed to such parasites (34, 35). The BIS includes a series of cognition and behaviors which involve detecting pathogens perceptually, evaluating the threat of infection, and initiating avoidance behavior (36). However, the underlying psychological basis of the BIS has not been clarified (37). Previous studies explored the underlying mechanism from different aspects of cognition, such as basic visual perception, tactile sensitivity, and other basic conditions (37–39). The BIS has been employed to explain the simple emotion of disgust that is directly related to disease (40) as well as the prejudices against elderly individuals (41), obese individuals (42), and individuals with physical disabilities (43). The emotion of disgust associated with eating insects and the perception that unprocessed insects are more unacceptable to eat than processed ones led us to consider the possibility that resistance to eating insects may be relevant or result from the functions of the BIS. People may feel nervous or disgusted when thinking about consuming insects. This is possibly because they associate insect food with parasites or other pathogens related to insects, which cause diseases and are potential risks to their health^1^.

The aim of the present study is to explore that whether the revulsion at eating insects is due to the BIS. Firstly we conducted a questionnaire survey to examine the experience, attitudes and concerns about insect food, non-insect food, and pathogens. Furthermore, we explored the semantic associations between insect food and non-insect food, between insect food and pathogens, and between non-insect food and pathogens by a picture-based rating experiment. We hypothesized that the attitudes toward pathogens, insect food, and food are significant predictors of the semantic associations between insect food and general food, and between insect food and pathogens.

## Method

### Ethics Statement

Approval for the study was obtained from the Ethics Committee for Psychological Studies at the Institute of Decision Science for a Sustainable Society, Kyushu University, Japan (No. 2017/2-2). All the methods employed were conducted in accordance with the relevant guidelines of the ethics committee. Each participant provided informed consent at the beginning of the survey.

### Participants

Of the 1,500 people we recruited online through Yahoo! Crowdsourcing service for this study, 1,478 respondents completed the experiments. A further 15 respondents did not answer more than one question and 94 rated all the questions the same. Consequently, these were considered deficient data and excluded from the data analysis. Thus, data collected from 1,369 (581 females and 788 males, mean age = 43.41 years, *SD* = 10.44 years) participants were analyzed for this study. These respondents were registered Yahoo! Crowdsourcing users, and they were randomly collected from all prefectures of Japan. All the respondents joined the survey online by using the internet browsers installed in their own devices, which included computers, tablet computers, and smartphones. We paid 15 T-points, which equaled 15 Japanese Yen to each of the respondents who have accomplished all the experimental trials via Yahoo! Crowdsourcing.

### Procedure and Materials

The survey comprised three phases and was conducted in Japanese. In the first phase, simple notices were presented at first to inform that the survey consisted of a questionnaire task and a rating task on food (including insect food) and pathogens, that the participants could withdraw from the survey at any time if they felt uncomfortable, and that the obtained data would be only used for academic purpose. After these notices, informed consent and demographic data, namely, age, and sex were collected. A questionnaire on food, entomophagy, and pathogens was administered in the second phase. During the third phase, a psychological experiment that involved rating semantic relations among visual stimuli of insect foods, non-insect foods, and pathogens was conducted. When a potential participant responded, the three phases were conducted sequentially. The time allocated for reading questions, observing stimuli, and giving responses was unlimited. The participants were not assessed in relation to the time it took them to complete the survey.

The translated version of the questions of the second phase is presented in Table 1. The *Food, Entomophagy*, and *Pathogen* categories comprised five, five, and four questions, respectively. These questions were created by authors originally for this study, based on informal interviews with students at Kyushu University, about their consideration, impression, or experience about insect food. Concerns about health/nutrition (Items 2, 7), taste/mouthfeel (Items 3, 9), and safety/hygiene (Items 4, 8) were asked commonly in both *Food* and *Entomophagy* categories, because they were considered as important factors in deciding whether or not to eat insect food. Other opinions which related to the encouragement to consume insect food, such as the willingness to try new food (Item 1), the environmental concerns about food production (Item 5), and the experience of eating insect (Item 6) were also incorporated to the questionnaire, with a direct inquiry about the willingness of eating insect food (Item 10). *Pathogen* category included intellectual comprehension about (Item 11) and emotional attitude to (Item 12) pathogens, experience of contact with pathogens (Item 13), and a direct question about concerns of the relationship between food and pathogens (Item 14). The 14 questions were assessed by means of a 5-point Likert scale, ranging from strongly disagree to strongly agree, from left to right on the iPad screen. No values or numbers were displayed on the screen. The response data were converted to values from 0 (strongly disagree) to 4 (strongly agree) automatically when they were uploaded to the server. Instructions were displayed at the beginning of the second phase and before the questions of each category.

**Table 1:** The English version of the questionnaire.

**Items**		**Abbreviation**	***M***	***SD***
**Food category**				
1	I like new food.	Food_neophilia	2.17	0.92
2	I keep the nutritional balance of diet in mind.	Food_nutrition	2.41	0.91
3	I am particular about the taste and mouthfeel of food.	Food_taste	2.69	0.78
4	I consider that safety and hygiene of food are important matters.	Food_safety	3.16	0.69
5	I take notice of the relationship between food and environment.	Food_environment	2.19	0.86
**Entomophagy category**				
6	I have more experiences in eating insect food than my friends.	Insect_experience	0.47	0.85
7	I think that insect food is healthy.	Insect_health	1.27	0.96
8	I am concerned about the safety and hygiene of insect food.	Insect_safety	2.97	1.10
9	I think that insect food is delicious.	Insect_taste	0.85	0.90
10	I would like to eat insect food.	Insect_willing	0.61	0.88
**Pathogen category**				
11	I am familiar with pathogens.	Pathogen_knowledge	0.99	0.88
12	I am scared of pathogens.	Pathogen_phobia	3.15	0.80
13	I am vulnerable to infections.	Pathogen_infection	1.75	0.86
14	I attend to the relationship between food and pathogens.	Pathogen_food	2.15	0.94

*The original version, which was used in the surveys, was in Japanese. Fourteen questions, classified in three categories, were tested in the surveys. The average values of the answers to the questions and their standard deviations are also presented in this table (n = 1,369)*.

**Figure 1:**
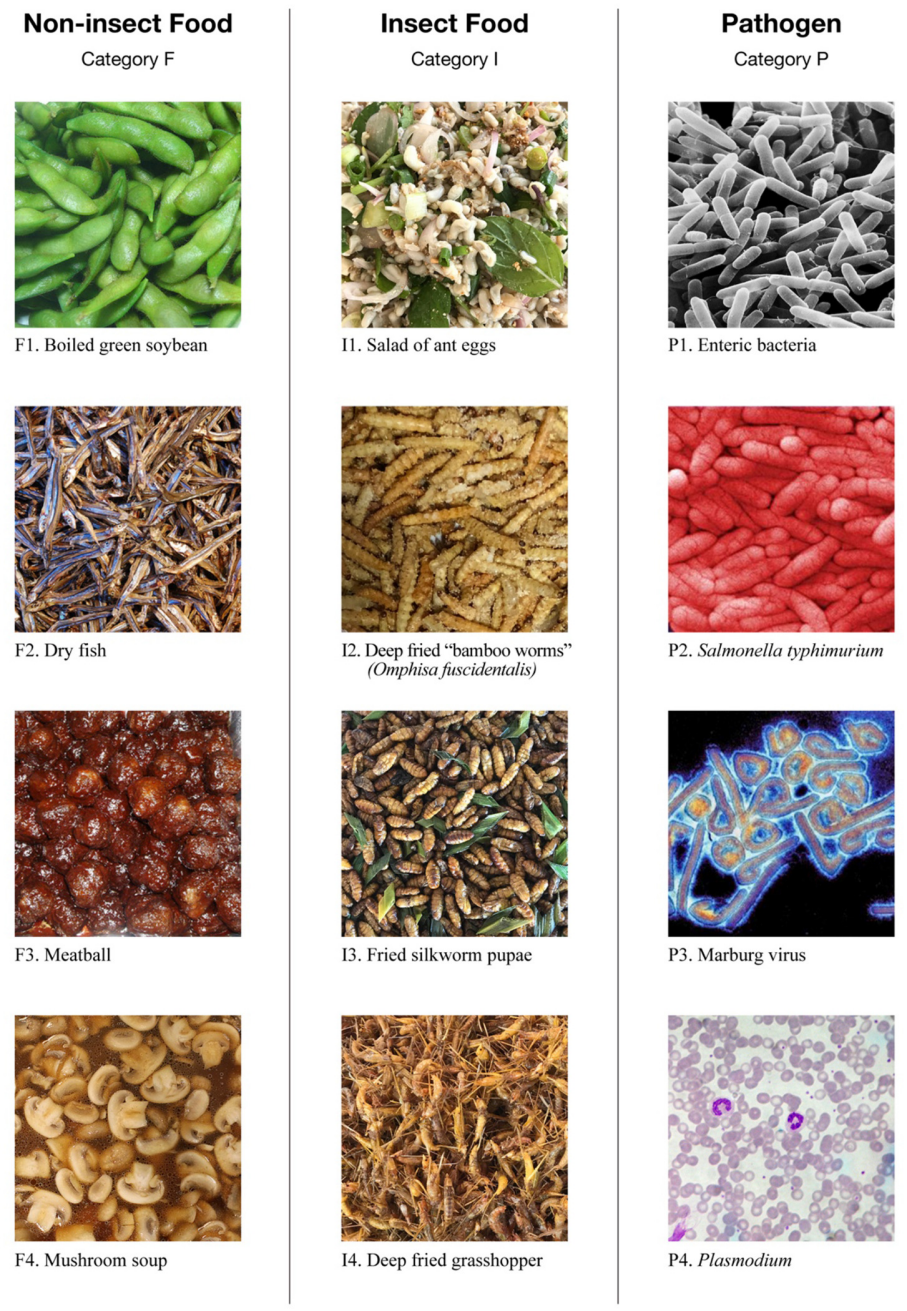
Image stimuli used in the experiment phase of the study. Twelve pictures in three categories were employed in the experiment.

**Figure 2:**
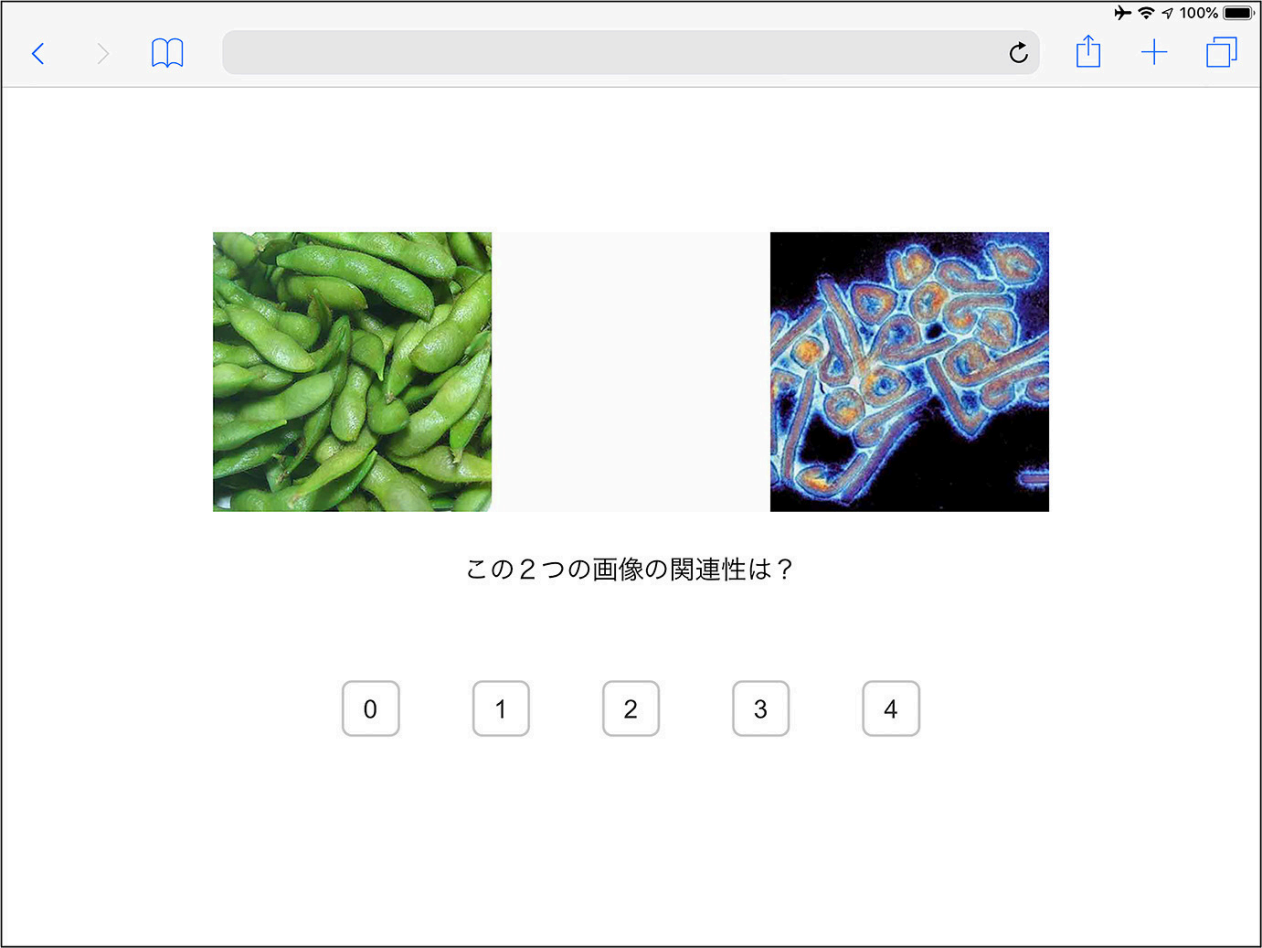
Screenshot of one trial in the experiment demonstrated by Apple iPad Air 2. The experiment was conducted in Japanese. The Japanese sentence in the center of the display means, “*How are the two pictures conceptually related to each other*”.

In the final phase, the psychological experiment, the 12 pictures depicted in Figure 1 were employed as stimuli. The pictures were classified into three categories, each of which contained four pictures: *Non-insect food (Category F), insect food (Category I)*, and *pathogen (Category P)*. The pictures in category I were sampled by the author in field surveys in Laos (I1, February 2017) and Thailand (I2, I3, and I4, December 2017). The pictures in categories F and P were selected from the image database of ImageNet (44). We selected the pictures based on following criteria: Category F included four different types of foodstuffs, beans (F1), fish (F2), meat (F3), and edible fungi (F4), with four different types of cooking/consuming methods, boiling (F1), drying (preserved food, F2), stewing (F3), and soup (F4). All these foodstuffs and cooking methods are common in Japanese diet and are consequently easy to be recognized by Japanese participants. Category I employed pictures of insect food which covered the four typical metamorphoses of insect, eggs (I1), larvae (I2), pupae (I3), and imagoes (I4). Category P included pictures of three typical infectious agents, bacteria (P2), virus (P3), and parasites (P4), with one picture of enteric bacteria (gut flora, P1) which is not disease-causing normally. Participants could understand that these pictures were about insect food or pathogens, because the notices at the beginning of the survey have mentioned. Pairs of pictures in different categories were displayed simultaneously. The participants were asked to rate how the two pictures were related to each other by selecting a value from 0 to 4, which indicated weak to strong semantic relations, respectively. One picture was paired with eight pictures in the other categories. Thus, a total of 96 pairs of pictures were generated as stimuli. A screenshot of one trial in this phase is illustrated in Figure 2 (a demonstration on Apple iPad Air 2). The size of each picture was 500^*^500 pixels. The two pictures in a pair were aligned vertically, with a horizontal distance of 500 pixels. The stimuli set with two pictures was located at the vertical center line (invisible) of the display. The question, “*How are the two pictures conceptually related to each other?”* as well as the value options were displayed below the stimuli set. Each pair of pictures was tested twice in a random order. Thus, a total of 192 trials were conducted in the experiment phase. Besides the 192 trials, at the beginning of this phase, five practice trials with random stimuli set were conducted to help participants get accustomed to the rating task. After all the three phases were accomplished, some additional information was displayed to help the respondents obtain rewards.

### Data Analysis

The rated data of semantic relations between the pictures were compressed by calculating the mean values of picture pairs in the same categories. Consequently, the data of 96 picture pairs were summarized into three representative values of the semantic relationships between non-insect food and insect food (F-I pair), between non-insect food and pathogen (F-P pair), and between insect food and pathogen (I-P pair). We conducted a one-way within-participant analysis of variance (ANOVA) on the mean rated semantic relationship with the factor of the category pair, and multiple comparisons based on Tukey's method by using R (Version 3.4.3 GUI 1.70 El Capitan build).

To clarify whether the participants' rating of semantic relations among pictures of insect food, non-insect food and pathogens could be significantly predicted by their attitudes toward food, entomophagy, and pathogens, we also conducted a series of multiple linear regression analyses by employing IBM SPSS Statistics Base (Version 25). The mean rated values on the semantic relationships of the F-I, F-P, and I-P pairs were considered dependent variables. For each of the three analyses, the mean scores of the questions on the related categories were used as explanatory variables (e.g., the 10 questions in the *Food* and *Entomophagy* categories were explanatory variables for the semantic relationship of the F-I pair). All independent variables were used in the models.

Basically, we used SPSS Statistics for data analysis. However, we used R to conduct one-way within-participant ANOVA because this analysis was not available in our SPSS (Statistics Base Version 25).

### Pilot Survey

We conducted a pilot survey by employing 20 students at Kyushu University to check the validity of the question items and the pictorial stimuli, to test the performance of the program, and to try the analytical methods with pilot data set. Details of the pilot survey were provided as Supplementary Materials.

## Results

**Figure 3:**
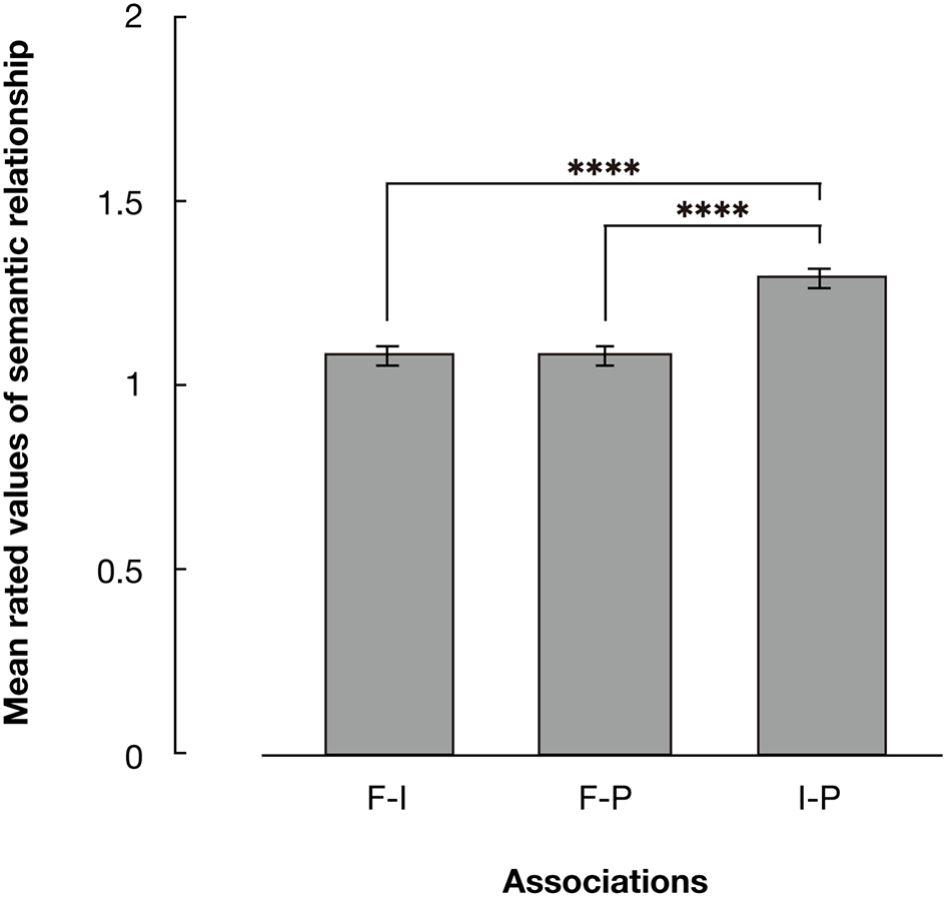
The results of the experiment phases (*n* = 1,369). Rated values of semantic relations between pictures were summarized by category. Error bars denote standard errors of the mean. Asterisks indicate significant differences resulting from multiple comparison tests for the main effect of category pairs; ****p < 0.001.

The mean scores of the questionnaire survey are displayed in Table 1. The summarized values of N-F, N-P, and I-P pairs are depicted in Figure 3. The results of a one-way within-participant ANOVA revealed a significant main effect of the picture pair [*F*_(2, 2, 736)_ = 113.319, *p* < 0.001 partial η^2^ = 0.077]. Multiple comparisons based on Tukey's method revealed significant differences between the N-F and I-P pairs, and between the N-P and I-P pairs (*p*_s_ < 0.001). The mean rated semantic relationship between insect food and pathogens were significantly higher than those between non-insect food and pathogens, and between non-insect food and insect food.

The results of multiple linear regression analyses revealed that the regression equation was significant when the dependent variable was F-I [*F*_(9, 1, 358)_ = 6.320, *p* < 0.001; *R*^2^ = 0.044], F-P [*F*_(9, 1, 358)_ = 4.773, *p* < 0.001; *R*^2^ = 0.031], or I-P [*F*_(9, 1, 358)_ = 4.994, *p* < 0.001; *R*^2^ = 0.032]. Details of the regression analyses are presented in Table 2. Concerns on safety and the environmental relationship of food were found to be significant predictors of the semantic links of insect and non-insect food. However, considering insect food to be healthy became a negative predictor. Furthermore, the experience of consuming insect food was a significant predictor of the F-I association. In relation to the I-P association, we revealed that the experience of consuming insect food and the attention given to the relationship between food and pathogens were significant positive predictors. Furthermore, viewing insect food as healthy was a significant negative predictor. The correlations among the questionnaire items were also tested (Table 3). Because of the large number of samples, significant correlations were found in most of the pairs. There were some strongly significant correlations that implied the relationship between the revulsion at entomophagy and the BIS. For example, the negative correlations of the willingness to eat insect food with the concerns about food safety, hygiene of insect food, and phobia about pathogens (*p*_s_ < 0.001).

**Table 2:** Results of multiple linear regression analyses (*n* =1,369).

	**F-I**			**F-P**			**I-P**		
**Variable**	***B***	***SE B***	**β**	***B***	***SE B***	**β**	***B***	***SE B***	**β**
Food_neophilia	0.02	0.03	0.02	0.04	0.03	0.04			
Food_nutrition	−0.03	0.03	−0.04	−0.03	0.03	−0.03			
Food_taste	0.02	0.03	0.01	−0.08	0.04	−0.07^*^			
Food_safety	−0.08	0.04	−0.06^*^	−0.06	0.04	−0.05			
Food_environment	0.12	0.03	0.12^***^	0.11	0.03	0.10^**^			
Insect_experience	0.14	0.03	0.14^***^				0.10	0.04	0.09^**^
Insect_health	−0.09	0.03	−0.10^**^				−0.13	0.03	−0.13^***^
Insect_safety	−0.03	0.02	−0.04				0.03	0.03	0.03
Insect_taste	−0.04	0.04	−0.04				0.01	0.05	0.01
Insect_willing	0.06	0.04	0.06				0.00	0.05	0.00
Pathogen_knowledge				0.01	0.03	0.01	0.00	0.03	0.00
Pathogen_phobia				−0.03	0.03	−0.02	−0.02	0.04	−0.02
Pathogen_infection				0.05	0.03	0.05	0.04	0.03	0.04
Pathogen_food				0.08	0.03	0.08^*^	0.12	0.03	0.12^***^
*R*^2^		0.04			0.03			0.03	
*F*	6.32^***^			4.77^***^			4.99^***^		

*The mean rated values of the semantic relationship of category pairs were dependent variables and the mean scores of the questions of the related categories were explanatory variables. All independent variables were employed in the models*.

*** *p < 0.001*,

** *p < 0.01*,

* *p < 0.05*.

**Table 3:** Correlations between mean results of questionnaire items.

		**1**	**2**	**3**	**4**	**5**	**6**	**7**	**8**	**9**	**10**	**11**	**12**	**13**	**14**
1	Food_neophilia	1.00													
2	Food_nutrition	0.17^***^	1.00												
3	Food_taste	0.33^***^	0.28^***^	1.00											
4	Food_safety	0.10^***^	0.33^***^	0.37^***^	1.00										
5	Food_environment	0.21^***^	0.39^***^	0.31^***^	0.33^***^	1.00									
6	Insect_experience	0.08^**^	0.00	−0.04	−0.09^***^	0.12^***^	1.00								
7	Insect_health	0.04	0.05^*^	0.03	−0.03	0.14^***^	0.37^***^	1.00							
8	Insect_safety	0.05^*^	0.10^***^	0.13^***^	0.25^***^	0.07^**^	−0.18^***^	−0.07^**^	1.00						
9	Insect_taste	0.09^***^	0.03	−0.03	−0.05^*^	0.16^***^	0.50^***^	0.59^***^	−0.16^***^	1.00					
10	Insect_willing	0.08^**^	−0.02	−0.03	−0.12^***^	0.14^***^	0.50^***^	0.52^***^	−0.20^***^	0.75^***^	1.00				
11	Pathogen_knowledge	0.10^***^	0.16^***^	0.11^***^	0.05^*^	0.27^***^	0.26^***^	0.14^***^	−0.03	0.21^***^	0.17^***^	1.00			
12	Pathogen_phobia	−0.01	0.06^**^	0.09^**^	0.30^***^	0.06^*^	−0.20^***^	−0.07^**^	0.37^***^	−0.16^***^	−0.20^***^	−0.12^***^	1.00	
13	Pathogen_infection	0.00	0.06^*^	0.03	0.04	0.09^**^	0.07^**^	0.07^**^	0.09^***^	0.02	0.04	0.10^***^	0.15^***^	1.00	
14	Pathogen_food	0.11^***^	0.34^***^	0.20^***^	0.28^***^	0.47^***^	0.07^**^	0.10^***^	0.20^***^	0.12^***^	0.08^**^	0.33^***^	0.18^***^	0.21^***^	1.00

*** *p < 0.001*,

** *p < 0.01*,

* *p < 0.05*.

*Asterisks indicate significant correlations*.

## Discussion

In this study, to explore whether the BIS influenced the acceptability of eating insects, we tested the semantic associations between non-insect food and insect food, between insect food and pathogens, and between non-insect food and pathogens by an image-based rating experiment. This was followed by a questionnaire on food, entomophagy, and pathogens. The test of semantic associations revealed simple but clear results, namely, the association between insect food and pathogens (I-P association) were significantly stronger than the associations between the other two pairs. This implies that the participants associated insect food with pathogens more than non-insect food. We are of the view that this result corresponds with the general decline of entomophagy in Japan in the context of the BIS. In comparison to non-insect food, people associated pathogens significantly more with insect food. Accordingly, they believed that insect food is not appropriate to eat because of concerns about food safety and hygiene. The results of regression analyses supported this finding. The vulnerability of infections and self-reported attention to pathogens were revealed as significant positive predictors of the I-P association. In contrast, thinking insect food is healthy negatively affected the I-P association. This implies that when people perceive that insect food is healthy, they will associate insect food with pathogens less. Concerns about health also suggested the mediator function of the BIS.

In contrast to the I-P association, the association between non-insect food and insect food (F-I association) suggested the level that insect food is accepted as food because a stronger F-I association implies a weaker distinction between insect food and non-insect food. Some evidence of the BIS-based hypothesis was found in the regression analyses. For example, concerns about food safety were revealed as significant negative predictors of the F-I association. People who are concerned about food safety revealed significantly less F-I association. This implies that people who are concerned about food safety have a lower level of acceptance of insect food as food. Off the context of the BIS, we found several domains such as taste and environmental concerns, which contributed to the F-I association. It appears that these two factors can be employed to promote eating insects so as to enhance the relationship between insect food and non-insect food. This study also revealed that adaptation is important in the behavior related to eating insects. Previous research found that people are willing to consume processed insect products and those who have consumed processed insect products are more willing to consume unprocessed ones (28). In our experiment, the results of regression analyses demonstrated the experience of eating insects as a significant positive predictor of the F-I association. This suggests that the more people consume insect food, the more insect food is likely to be classified as general food.

The results of this study provided informative implications to the marketing of insect food products, and to the policy making of insect food promotion. The activation of the BIS induces revulsion at eating insect food. Thus, weakening the association between insect food and infectious risk is considered as an effective way to reduce the revulsion. Emphasizing safety and hygiene with transparent production and processing of insect food, using insect-based ingredient, such as powders or paste instead of “visible” insects to decrease the association between “insect” and “parasite,” or establishing a safety certification system for insect food are all beneficial for building a sanitary and hygienic image of insect food. Furthermore, it was suggested that experience of consumption would help customers adapt to insect food. Thus, increasing the exposure of insect food, and providing chance to try them will help develop new potential customers.

The present study is subject to limitations, which require further investigation in future. Firstly, the regression analyses revealed some results, which were difficult to explain. For example, for the F-I associations, thinking insect food was healthy was a significantly negative predictor. This implies that people who consider insect food to be healthy had fewer F-I associations. This result should be carefully verified in future studies. Secondly, the reliability of the items used in our questionnaire need further validation, because they were originally created based on interviews, and were all single statements for each factor. Based on the results of the present study, the development of reliable scales to explore the relationship between entomophagy and the BIS is necessary in future. Lastly, even though we collected considerably large samples in the main study by conducting an online survey, data from those who could not participate in online survey were not included. Considering the limitations of our methodology, it is recommended future studies conduct larger-scaled surveys with more diverse demographics, by using other experimental methods testing the semantic associations, such as an implicit association test. Global-scaled and longitudinal study is also recommended to reveal the difference and changes of the BIS on entomophagy across genders, generations, and cultures.

## Data Availability Statement

The datasets generated for this study are available on request to the corresponding author.

## Author Contributions

KQ and YY conceived and designed the study. KQ conducted the experiments, performed the statistical analysis, and wrote the first draft of the manuscript. YY drafted the manuscript and made critical revisions. Both authors read and approved the submitted version.

## Conflict of Interest

The authors declare that the research was conducted in the absence of any commercial or financial relationships that could be construed as a potential conflict of interest.

## Footnotes

^1^Coincident with the comment from reviewer, we consider that this association occurs not only in insects, but also in other animals which are not regarded as food commonly. This association is supposed to be related to arousal of the cognition that consuming such things will cause disadvantage somehow. Rather than insects, other animals which were scavengers or living in insanitary environment, such as crows and bats are also difficult to be accepted as food. Investigation of the BIS on other inedible animals is an open question for studies in future.
