# Supplementary material for: Exploring the Role of the Behavioral Immune System in Acceptability of Entomophagy Using Semantic Associations and Food-Related Attitudes
Source: Front Nutr. 2020 May 20;7:66. doi: 10.3389/fnut.2020.00066 (PMC7251053; doi:10.3389/fnut.2020.00066)
Supplement: Supplementary file 1 [file Data_Sheet_1.pdf]

Supplementary Material

1 Details of the pilot survey

1.1 Participants

The pilot survey included 20 Japanese participants (12 females and 8 males, mean age = 24.50 years, *SD* = 4.38 years). All participants were students or administration staffs at Kyushu University, and were recruited directly by the authors. They were all residents of Fukuoka city, representing the population of relatively young urban citizens of Japan. Their participation was voluntary, and they were not informed of the purpose of the study before participating in the survey. The purpose of the study was revealed after the survey to the participants who have inquired. They were informed that they could withdraw from the study at any stage if they felt uncomfortable with items in the questionnaire and/or the stimuli in the experiment. Furthermore, the participants were not remunerated for their participation.

1.2 Apparatus, Procedure, Materials and Data Analysis

Different with the main study in which data were collected online, the participants of the pilot survey used tablet computers (Apple, iPad Air 2, A1567) to complete the survey. Eight iPads were prepared to make it possible for more than one participant to complete the survey simultaneously and independently. The model, specification, purchase lot, and operating system (iOS 11.2.6) of each iPad was identical. The survey was programmed and conducted by jsPsych (de Leeuw, 2015). The participants used Safari, which had been pre-installed in the iPad, to complete the survey. The participants were required to view the display of their iPad (9.7-inch LED-backlit Multi-Touch display

with IPS technology, 2048*1536 pixels at 264 ppi) binocularly at a convenient viewing distance and to answer the questions by touching the display. All the data obtained were automatically uploaded to our server at the end of the survey. Procedure, materials and data analysis of the pilot survey were identical to those of the main study.

1.3 Results

The mean scores of the questionnaire were calculated and shown in Supplementary Table 1. Representative values of semantic relationships between picture categories are depicted in Supplementary Figure 1. The results of ANOVA revealed a significant main effect of the category pair ($F_{2, 38}$ = 3.472, p = 0.041, partial η^2 = 0.155). Although multiple comparisons based on Tukey's method showed that there was no significant difference between any of the two category pairs (ps > .05), the relation between non-insect food and pathogen (F-P) appeared to be rated weaker than the other two pairs (F-I and I-P).

The results of multiple linear regression analyses revealed that the regression equation was significant when the dependent variable was F-I ($F_{10,9}$ = 6.588, p = 0.005; R^2 = 0.880) or I-P ($F_{9,10}$ = 4.539, p = 0.013; R^2 = 0.803), but not significant with the dependent variable of F-P ($F_{9,10}$ = 0.377, p = 0.921; R^2 = 0.254). Details of the results of the three regression analyses are presented in Supplementary Table 2. The concerns about nutrition, taste, safety, and environmental relationships of food as well as considering insect food to be healthy, safe, or tasty were revealed as significant predictors of the semantic links of insect and non-insect food. In relation to the semantic links between insect food and pathogens, the willingness to consume insect food and vulnerability to infections were revealed as two significant predictors. The correlations among the questionnaire items were also tested and regression analyses performed. Details of the correlations are presented in Supplementary Table 3.

1.4 Discussion

The results of regression analyses provided reliable evidence for our hypotheses, that some attitudes toward food, insect food, and pathogens are predictors of the semantic associations between insect food and general food, and between insect food and pathogens. The participants who were concerned with the nutritional balance, taste, and environmental issues of food, and who also considered insect food to be healthy and tasty revealed stronger associations between the pictures of insect and non-insect food. The aspects of nutrition, health, and environment concur with the well-known advantages of insect food. Taste was also clarified as a factor, which may contribute to the promotion of insect food. In contrast, concerns about food safety and hygiene for both insect and non-insect foods negatively affected the associations between insect and non-insect food. This result implied a possible activation of the BIS when people considered insects as food because food safety was more prevalent. In relation to the semantic associations between insect food and pathogens, the willingness to consume insect foods revealed a negative effect. In other words, the participants who accepted insect food did not associate insect foods with pathogens. This implies that the function of the BIS was weakened by a strong willingness to eat insects. This result coincides with other unique but robust dietary traditions, which are dangerous to one's health, but cannot be prevented by BIS. In southern China, millions of people have been infected by Chinese liver fluke (*Clonorchis sinensis*), a serious and fatal parasite, because of the robust dietary habit of consuming raw clearwater fish (Zhang et al., 2007). On the other hand, the participants who considered themselves vulnerable to infections showed stronger associations between insect food and pathogens. This may have been caused by a more sensitive attitude toward foods, which were associated with pathogens, based on the BIS.

The analyses of correlations also revealed some interesting results. The significant positive correlations related to food neophilia suggested that people who are willing to try new food have a

more positive attitude to insect food because they may have experience of consuming insect food and may consider insect food to be tasty. Those who were concerned about the nutritional balance and safety of food are likely to consider insect food to be unhealthy and inedible whereas those who believed insect food is healthy may be willing to consume such and think it is delicious. However, some of the results were mystifying such as the positive correlations of consuming insect food with phobias and vulnerability to pathogens.

The semantic relationship between non-insect food and pathogen (F-P) was rated lower than the relationships between non-insect and insect food (F-I), and between insect food and pathogen (I-P). The results imply that insect food was associated more with pathogens in comparison to non-insect food. However, due to the lack of significance, further investigation is necessary to clarify the difference.

The results of the pilot survey provided evidence that the unwillingness to consume insect food was based on the BIS. However, the participants of the pilot survey were all recruited in university, who could only represent a limited part of the population in Japan. To explore the semantic relationship between the different image categories and to clarify the mystifying results of the correlation analyses, a further study with more diverse samples and more statistical power was crucial. Consequently, we conducted an online-based survey as our main study to collect larger-scaled data.

2 Reference

De Leeuw, J. R. (2015). jsPsych: A JavaScript library for creating behavioral experiments in a Web browser. *Behav. Res. Methods* 47(1), 1-12.

Zhang, R., Gao, S., Geng, Y., Huang, D., Yu, L., Zhang, S., Cheng, J., & Fu, Y. (2007). Epidemiological study on Clonorchis sinensis infection in Shenzhen area of Zhujiang delta in China. *Parasitol. Res.* 101(1), 179-183.

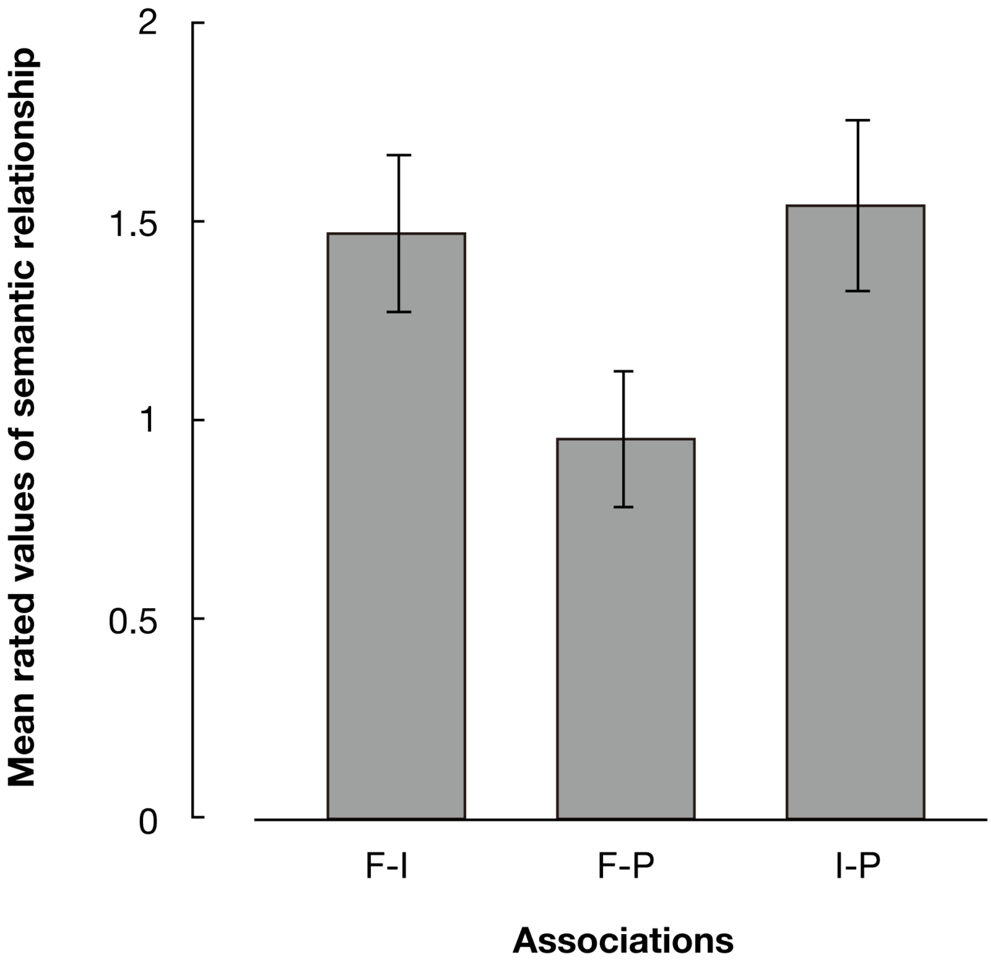

Supplementary Figure 1. The results of the experiment phases in the pilot study (n = 20). Rated values of semantic relations between pictures were summarized by category. Error bars denote standard errors of the mean.

Supplementary Table 1. The average values of the answers to the questionnaire and their standard deviations in the pilot study (n = 20).

Items		Abbreviation	*M*	*SD*
Food category				
1	I like new food.	Food_neophilia	2.60	1.14
2	I keep the nutritional balance of diet in mind.	Food_nutrition	2.45	1.19
3	I am particular about the taste and mouthfeel of food.	Food_taste	3.05	0.83
4	I consider that safety and hygiene of food are important matters.	Food_safety	3.35	0.59
5	I take notice of the relationship between food and environment.	Food_environment	2.30	1.13
Entomophagy category				
6	I have more experiences in eating insect food than my friends.	Insect_experience	1.05	1.19
7	I think that insect food is healthy.	Insect_health	1.95	1.15
8	I am concerned about the safety and hygiene of insect food.	Insect_safety	2.75	1.33
9	I think that insect food is delicious.	Insect_taste	1.10	0.97
10	I would like to eat insect food.	Insect_willing	1.25	1.25
Pathogen category				
11	I am familiar with pathogens.	Pathogen_knowledge	0.90	0.91
12	I am scared of pathogens.	Pathogen_phobia	3.20	0.70
13	I am vulnerable to infections.	Pathogen_infection	1.45	1.15
14	I attend to the relationship between food and pathogens.	Pathogen_food	2.10	1.12

Supplementary Table 2. Results of multiple linear regression analyses in the pilot study.

	F-I				F-P			I-P			
Variable	*B*	*SE B*	*β*		*B*	*SE B*	*β*	*B*	*SE B*	*β*	
Food_neophilia	0.03	0.16	0.04		0.04	0.20	0.06				
Food_nutrition	0.33	0.14	0.45	*	0.19	0.26	0.29				
Food_taste	0.81	0.27	0.77	*	0.26	0.38	0.28				
Food_safety	-1.69	0.34	-1.14	**	0.16	0.51	0.12				
Food_environment	0.29	0.13	0.38	*	-0.25	0.33	-0.37				
Insect_experience	0.03	0.12	0.04					0.07	0.16	0.09	
Insect_health	0.74	0.17	0.97	**				0.32	0.17	0.38	
Insect_safety	-0.29	0.10	-0.44	*				-0.28	0.16	-0.40	
Insect_taste	0.43	0.19	0.47	*				0.05	0.22	0.05	
Insect_willing	-0.01	0.17	-0.01					-0.82	0.19	-1.07	**
Pathogen_knowledge					-0.11	0.45	-0.13	0.23	0.36	0.22	
Pathogen_phobia					0.03	0.40	0.03	0.17	0.33	0.12	
Pathogen_infection					0.05	0.30	0.08	0.45	0.18	0.54	*
Pathogen_food					0.05	0.55	0.07	-0.29	0.34	-0.34	
R^2		0.88				0.25			0.80		
F		6.59	**			0.38			4.54	*	

*** $p < .001$. ** $p < .01$. * $p < .05$.

Supplementary Table 3. Correlations between mean results of questionnaire items in the pilot study. Asterisks indicate significant correlations.

		1	2	3	4	5	6	7	8	9	10	11	12	13	14
1	Food_neophilia	1.00													
2	Food_nutrition	0.18	1.00												
3	Food_taste	0.19	0.57 **	1.00											
4	Food_safety	0.22	0.37	0.51 *	1.00										
5	Food_environment	-0.02	0.48 *	0.55 **	0.55 **	1.00									
6	Insect_experience	0.40 *	-0.02	-0.11	0.28	-0.05	1.00								
7	Insect_health	0.19	-0.41 *	-0.55 **	0.03	-0.31	0.08	1.00							
8	Insect_safety	0.00	-0.22	-0.08	-0.02	-0.02	0.04	0.34	1.00						
9	Insect_taste	0.51 *	-0.36	-0.40 *	0.12	-0.27	0.36	0.53 **	0.14	1.00					
10	Insect_willing	0.33	-0.57 **	-0.57 **	-0.27	-0.43 *	0.31	0.52 **	-0.12	0.59 **	1.00				
11	Pathogen_knowledge	0.01	0.19	0.43 *	0.36	0.44 *	-0.04	-0.31	-0.37	0.01	-0.12	1.00			
12	Pathogen_phobia	0.11	0.39 *	0.35	0.46 *	0.52 **	0.43 *	-0.25	-0.28	-0.11	-0.24	0.20	1.00		
13	Pathogen_infection	0.19	-0.16	0.20	0.38 *	0.09	0.45 *	0.02	0.11	0.38 *	0.10	-0.06	0.34	1.00	
14	Pathogen_food	0.12	0.36	0.62 **	0.67 **	0.73 ***	0.15	-0.32	-0.05	0.04	-0.36	0.73 ***	0.51 *	0.37	1.00

*** $p < .001$. ** $p < .01$. * $p < .05$.
